# Air and Water

**Published:** 1892-06-04

**Authors:** 


### The Doctor's Armchair.

### AIR AND WATER.

Man is a rational animal, no doubt, but not extremely rational when taken in the mass. At most of the London railway stations there are automatic standards for "trying your weight" and "taking your height," so that all of us who are in the habit of travelling may furnish ourselves with accurate information on these points without inconvenience and at the smallest possible cost. It would be an advantageous thing if similar trustworthy standards were available whereby we might try our mental weight and take our intellectual height. The information, though it might surprise and mortify some of us, could hardly fail of being serviceable; and if in addition to this knowledge for private use we could have the intellectual height and weight of all our public men ascertained and published at a cheap rate in the form of a carefully revised Year Book, a rapid growth of the higher civilisation might be looked for in a very short time.

These ideas are suggested by the reading of a little book on "Air and Water." The volume constitutes one of the University Extension series, and its author is Mr. Vivian B. Lewis, Professor of Chemistry, at the Royal Naval College, Greenwich. The particular passage which arrested attention, and started the writer off on a line of speculative reflection, was this: "For more than sixteen centuries after Aristotle's time, no important advance was made in the ideas existing with regard to the atmosphere; when at rest it was forgotten, when in motion it was considered a spirit. But with the rise of Galileo in the last years of the sixteenth century, air began to have a history." That the twenty centuries between Aristotle and Galileo should have produced no man whose curiosity and capacity were sufficient to prompt him to the making of scientific warfare upon the atmosphere, which is one of the most fundamental of all the conditions of animal life, is extremely surprising, and speaks volumes to him who has an ear.

The modern world, happily, has set out upon enterprises of discovery in all directions. Long before darkest Africa had its Livingstone and its Stanley, the mysterious atmosphere in which we all live had its Galileo, its Torricelli, its Priestley, and its Lavoisier. Men all breathed oxygen before Galileo taught them that the air was a material substance, and could be weighed, and before other experimenters showed them that animal life was impossible in a vacuum. But though they breathed air, and utilised it, and lived by it, they did not know, in a scientific sense, anything about it. That was discreditable to their intelligence, and so to a certain extent shameful. But their want of knowledge also had its practical disabilities. Their bodily health, for example, often suffered when it need not have done; their cattle, and in some measure their crops, experienced injury. They themselves were in the slavery of ignorance rather than in the freedom of knowledge; and so their lives were less happy and less consciously victorious. Our own generation, following in the footsteps of its immediate predecessors, is rapidly making up for the time lost during the centuries between Aristotle and Galileo. Those two scientific heroes number their competent followers by tens of thousands in this generation. At the universities of all countries are men of science who consider it greater honour to make new conquests in knowledge than any other fame which the world can confer upon them. In the class-rooms of those professors are hundreds of young men to whom scientific knowledge and study are a kind of delightful intoxication. The universities, fired with an ardour which is as noble as it is modern, spread themselves out in all directions, by means of their peripatetic lecturers, in order that every mother's son and daughter, of every class, who is endowed with curiosity and intelligence, may have at least a chance of becoming an Aristotle or a Galileo in some delightful feature. Best of all is the fact that the lectures given by the University extensionists are published in the form of cheap and convenient books: and if, as Carlyle says, "the true University is a library," then every youth and maiden who can save but so much as a few pence weekly, and lay them out in the purchase of well-chosen books, may in time acquire, in the best sense of the word, a University education, and be able, not only to guide himself, but to instruct his fellows.

That every man ought to be a craftsman, ought to have some kind of craft in his fingers whereby he can earn bread and butter, is freely admitted. But then he ought to be something of a philosopher as well, for if he be not, what is he better than the horse, or the cow, or the dog? The horse has his craft of ploughing and drawing carts; the cow has her craft of yielding milk, and permitting herself to be offered up by the butcher-priest on the altar of human nutriment; so, too, the dog has his craft of house-watching, and of carrying on a valorous and patriotic warfare against tramps. But the man, to be a man, must be something more than a mere craftsman. He must be capable of detaching his mind from the absorbing question of bread and butter, and looking at all things in heaven and earth with a sympathetic, and, as far as possible, a comprehending eye. The University extension series of text books offer easy ladders by which all classes of youthful readers and of those whose education has been limited may climb to knowledge, and to a reasonable judgment. This little volume on "Air and Water," is one of the most interesting and readable members of its class. The aim of the writer has been to make both philosophers and craftsmen. The practical information contained in the book is, as everyone may understand, of a kind that may be profitably utilised every day. Is it possible to imagine any householder who would not be materially advantaged from time to time by an extensive and intelligent knowledge of air and water? As a substitute for every kind of novel, except the very best, a little book like "Air and Water" has many advantages. It will be found most interesting even by uninstructed readers; and the mastery of knowledge is one of the best of tonics both for body and mind. For hospital nurses, for mothers of families, and even for medical students and practitioners, an interesting little work on such an important everyday topic is something to be thankful for.

